# High-resolution stereolithography: Negative spaces enabled by control of fluid mechanics

**DOI:** 10.1073/pnas.2405382121

**Published:** 2024-09-04

**Authors:** Ian A. Coates, William Pan, Max A. Saccone, Gabriel Lipkowitz, Dan Ilyin, Madison M. Driskill, Maria T. Dulay, Curtis W. Frank, Eric S. G. Shaqfeh, Joseph M. DeSimone

**Affiliations:** ^a^Department of Chemical Engineering, Stanford University, Stanford, CA 94305; ^b^Department of Mechanical Engineering, Stanford University, Stanford, CA 94305; ^c^Department of Radiology, Stanford University, Stanford, CA 94305

**Keywords:** microfluidics, negative-space, 3D-printing, stereolithography, vat-polymerization

## Abstract

Stereolithography enables the fabrication of three-dimensional (3D) freeform structures via light-induced polymerization. However, the accumulation of ultraviolet dose within resin trapped in negative spaces, such as microfluidic channels or voids, can result in the unintended closing, referred to as overcuring, of these negative spaces. We report the use of injection continuous liquid interface production to continuously displace resin at risk of overcuring in negative spaces created in previous layers with fresh resin to mitigate the loss of Z-axis resolution. We demonstrate the ability to resolve 50-μm microchannels, breaking the historical relationship between resin properties and negative space resolution. With this approach, we fabricated proof-of-concept 3D free-form microfluidic devices with improved design freedom over device material selection and resulting properties.

## Significance

Additive manufacturing has the potential to revolutionize conventional microfluidic fabrication processes by enabling three-dimensional (3D) design and production of complex geometries with enhanced functional components in a single manufacturing step. However, current tradeoffs between exposure energy and optical penetration depth of the print material limit resolution in stereolithography-based processes. We have developed, injection continuous liquid interface production (iCLIP) enabling the fabrication of high-resolution 3D microstructures not before possible through the control of fluid mechanics. iCLIP technology empowers the creation of freeform microfluidic devices, offering unparalleled 3D design freedom for design applications ranging from biomedical device engineering, microelectronic fabrication, and advanced separation sciences.

Negative spaces are crucial components of microfluidic devices, biomedical devices, vascular networks, separation media, and electronic circuits, enabling precise control of fluid flow, improved sensor accuracy, and enhanced separation efficiency (1–6). However, traditional fabrication methods such as photolithography, etching, and injection molding have proven to be time-consuming, expensive, and are limited to two-dimensional design (7, 8). Recently, additive manufacturing (AM) has emerged as a powerful alternative to these methods. AM enables the creation of complex three-dimensional (3D) structures with free-form geometries to integrate a wide range of functional elements (9–12). Furthermore, 3D-printed microsystems can be produced more rapidly and at a lower cost than conventional microsystem fabrication methods, making them an attractive option for research and industrial applications alike (13–15).

**Fig. 1.**
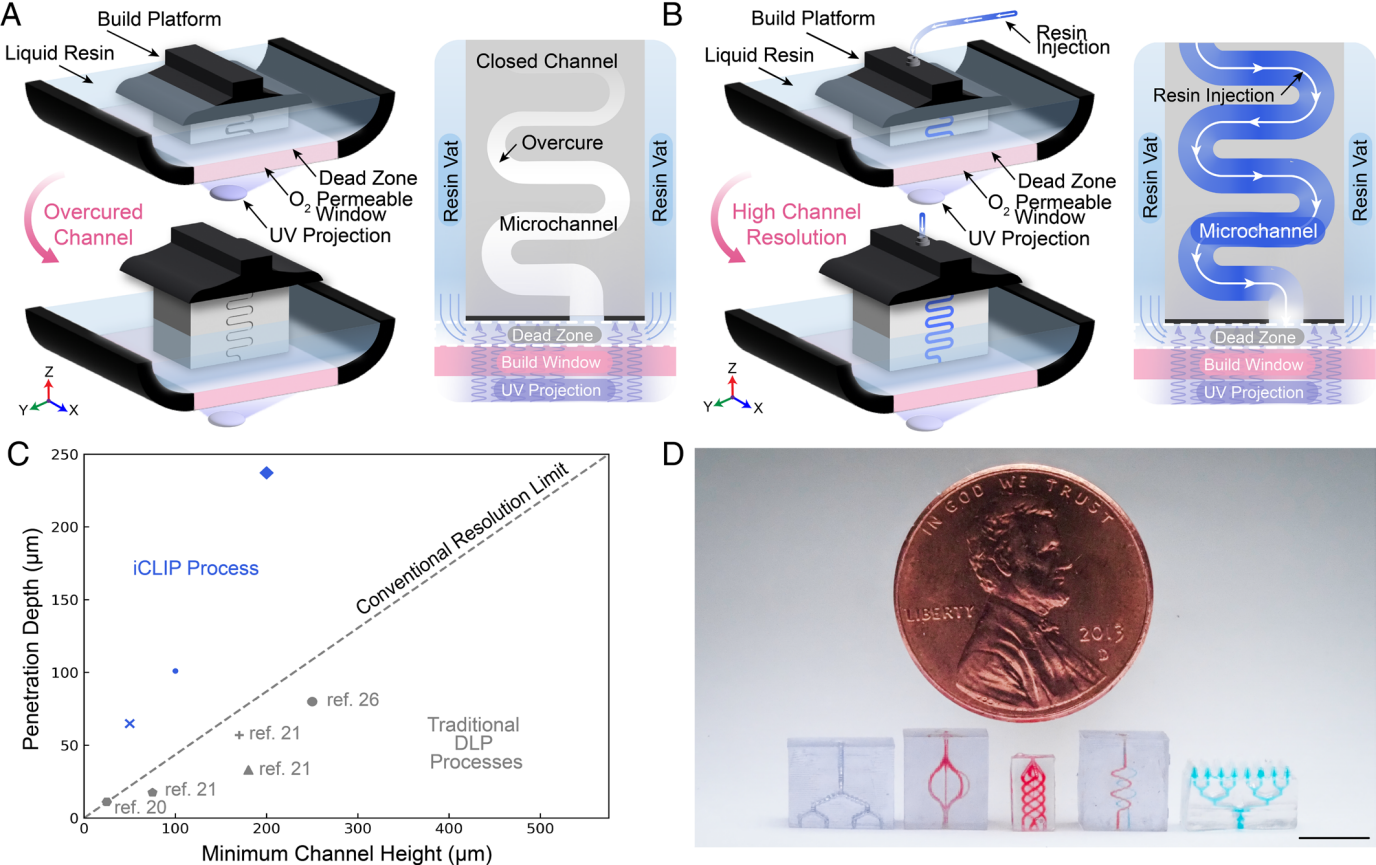
iCLIP enables fabrication of microscopic negative structures. (*A*) Schematic of CLIP process and the resulting overcuring effect. (*B*) Schematic of iCLIP process and the resulting resolved negative structures. (*C*) Relationship between UV light penetration depth and minimum channel height of iCLIP and other DLP systems (20–22). (*D*) Resulting microsystems including microfluidic distributer, vascular perfusion beds, and a microfluidic-enabled microarray patch printed via high-resolution iCLIP, with channels filled with dye for contrast. (Scale bar, 5 mm.)

Stereolithography has emerged as an attractive fabrication method among various AM processes due to its combination of scalability and high resolution. Among the stereolithography processes, digital light processing (DLP) has emerged as a promising AM technology for microsystem fabrication. Traditional DLP uses two-dimensional projections of ultraviolet (UV) light to cure layers of photopolymerizable resin layer-by-layer. Continuous liquid interface production (CLIP) is a related process which relies on resin renewal at the build surface through the creation of a continuous liquid interface—the dead zone—that enables resin to be drawn into the gap through suction forces created as the curing part is gradually pulled away from the window (Fig. 1*A*). The dead zone is created and maintained by a constant supply of oxygen—a polymerization inhibitor—that is fed through the highly oxygen-permeable window at the bottom of the resin reservoir (16). In stereolithography, light delivery is controlled by high-resolution optics that can precisely direct the UV light path to cure a single layer of resin in the XY-plane with high resolution (17, 18). However, achieving high resolution in the Z-axis, or the vertical direction, is more challenging. This is because it is difficult to confine the light completely to the layer being polymerized. Instead, UV light will exponentially decay beyond the intended layer, causing decreased part resolution in the Z-axis due to overcuring in previously created negative spaces (Fig. 1*A*) (16, 19). Specifically, the penetration depth (D_p_) of a resin represents the characteristic length at which exponential decay of intensity occurs within a resin. Historically, overcuring has limited the ability of stereolithographic processes to resolve negative spaces to only 2.3 times the characteristic penetration depth (20).

To address this issue, traditional approaches have attempted to confine the UV light in the Z-axis by incorporating UV light-attenuating additives into the liquid resins (1, 16, 21, 23). These additives absorb UV light, aiming to limit the polymerization to the desired layer thickness and improve the accuracy of the printed patterns. While the use of light-absorbing dyes or additives has shown some effectiveness, these dyes have certain drawbacks. They not only require higher light intensity to solidify the resin, which adds to fabrication time, but they also often possess toxic properties which restricts them in life sciences applications (24). Furthermore, the addition of these additives can leave fabricated parts optically opaque or translucent when transparent devices are often required. Therefore, finding alternative methods to enhance Z-axis resolution in stereolithography without relying on light-attenuating additives is crucial (5, 6, 25).

In the present study, we report the use of high-resolution injection CLIP (iCLIP) to achieve micrometer X, Y, and Z-axis resolution using synergistic control of high-resolution optical control and fluid mechanics. The iCLIP process platform was originally established to feed resin to the dead zone during printing to increase print speed and enable printing with higher viscosity resins and multimaterial printing (26). Our research extends the iCLIP system by continuously feeding a stream of fresh polymerizable resin through the build platform to displace trapped resin to preserve designed negative spaces and eliminate overcuring (Fig. 1*B*). Thus, the iCLIP process enables the fabrication of channels with significantly smaller heights/diameters than previously attainable. Using this method, we demonstrate the fabrication of microchannels with heights matching and exceeding a resin’s penetration depth (Fig. 1*C*). The possibility to fabricate high-resolution negative spaces using a greater variety of materials enables 3D-printing of high-resolution microsystem devices such as vascular beds and microfluidic-backed microneedles (Fig. 1*D*).

## Results

### Modeling Overcure.

In all DLP-based processes, including CLIP, XY resolution is restricted by the projected pixel size while Z-axis resolution is influenced by the penetration depth of a resin. The penetration depth is a critical parameter, unique to each resin, that determines how deeply UV light can penetrate the resin and initiate curing. A larger penetration depth leads to the accumulation of more UV light within the resin, which can unintentionally result in overcuring. Achieving Z-axis resolution comparable to XY-plane single-digit-micron features has been a challenge for researchers (20).

Using the Beer–Lambert law, E_N_, the cumulative UV-exposure energy per area after N exposures during part fabrication was determined. The equation for E_N_ is as follows:

[1] $$E_{N}=\sum_{n=0}^{N}I_{o}te^{-sn/D_{p}},$$

where n is the number of layers from the dead zone, I_0_ is the intensity of the UV irradiation at the dead zone, t is the UV exposure time, s is the layer slice thickness of each exposure, and D_p_ is the penetration depth determined by the resin’s material properties at the UV wavelength 385 nm (21, 27).

E_N_ depends on the resin’s D_p_, which governs the depth to which UV energy can penetrate and accumulate in negative spaces. The iCLIP system displaces trapped resin with fresh resin, maintaining a constant turnover that minimizes final E_N_ within microchannels, eliminating the need to reduce a resin’s D_p_ for better Z-axis resolution.

**Fig. 2.**
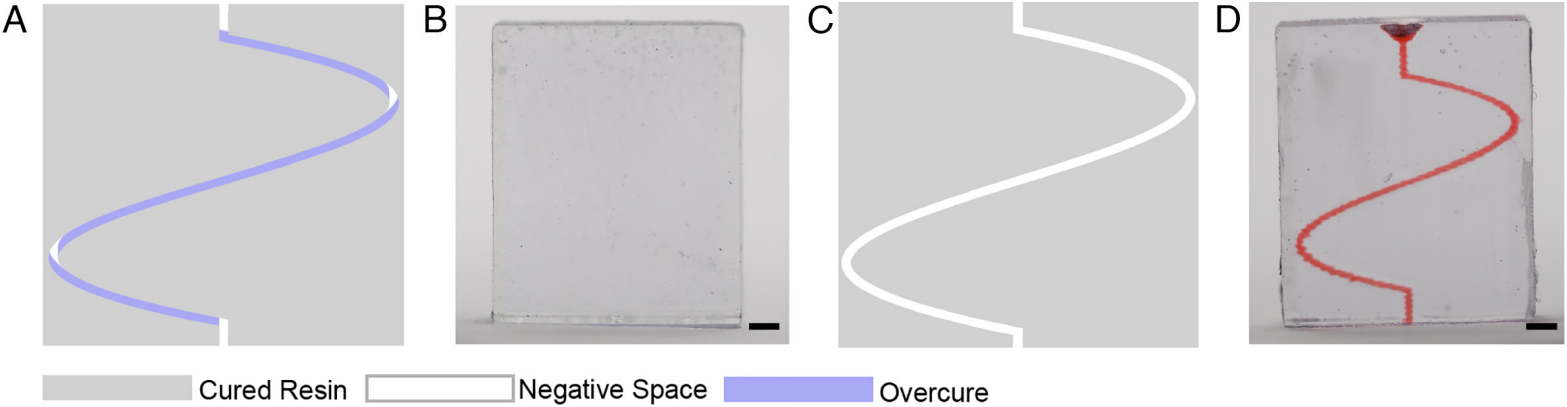
Comparing model and experimental overcuring effects between CLIP and iCLIP processes. (*A*) UV light accumulation in the CLIP process and the resulting modeled overcuring effect. (*B*) CLIP print result. (*C*) UV light accumulation in the iCLIP process and the resulting sinuous channel resolution. (*D*) iCLIP print result. (Scale bar, 1 mm.)

Eq. **1** enables us to predict UV dose accumulation within the entire 3D print microstructure. When the critical energy of trapped resin is exceeded, overcuring occurs (indicated by blue shading in Fig. 2*A*). Fig. 2 illustrates dose accumulation in a 200-μm serpentine microchannel during CLIP and iCLIP processes. Under CLIP conditions, without resin turnover, the accumulation model predicts overcuring that obstructs the microfluidic channel (Fig. 2*A*). The model is supported by the resulting CLIP print (Fig. 2*B*). Conversely, under iCLIP conditions, the continuous flow of fresh resin through the microchannel displaces trapped resin before it reaches the critical threshold, preserving the serpentine microchannel and mitigating overcuring (Fig. 2*C*). This is supported by the resulting iCLIP print (Fig. 2*D*).

### iCLIP Channel Preservation.

**Fig. 3.**
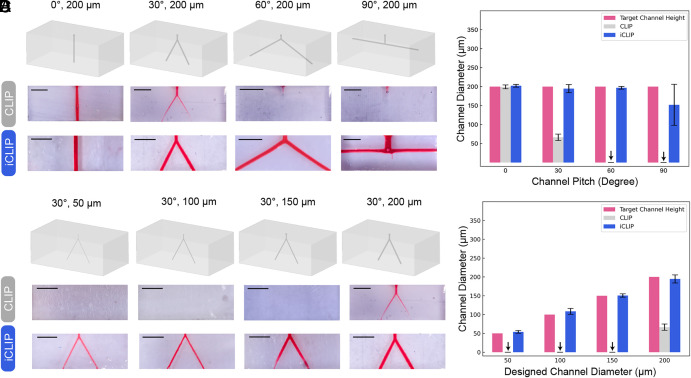
Mitigating overcuring in varying microfluidic channel geometries and sizes. (*A*) Resulting CLIP and iCLIP prints of varying channel pitches. (*B*) Resulting CLIP and iCLIP prints of varying channel diameters. (*C*) Evaluating resolution of varying channel pitch geometries using the CLIP and iCLIP systems. (*D*) Evaluating resolution of varying channel diameters using the CLIP and iCLIP systems. All scale bars 1 mm.

The ability of iCLIP to preserve the resolution of negative spaces in a variety of geometric configurations and channel resolutions was investigated. First, to evaluate the performance of iCLIP in resolving diverse microfluidic geometries, we designed microchannels with a diameter of 200 µm and varying pitch angles ranging from 0° to 90° (Fig. 3*A*). The 0° pitch channel, serving as the control, exhibits the least susceptibility to overcuring because it is never exposed to UV light beneath the fabricated channel as it is completely vertical. In contrast, the 90° pitch channel poses the highest risk of channel obstruction as the vertical Z-axis channel height decreases (*SI Appendix*, Fig. S3). Optical micrograph images of the cross-sectional profiles of the printed microchannels clearly demonstrate that iCLIP consistently achieves accurate resolution of channels, regardless of their pitch (Fig. 3 *A* and *B*). We do observe that the 90° pitch is resolved fully near the injection port while the channel further from the injection source is smaller in diameter. We hypothesize this is due to insufficient resin turnover before the channel becomes obstructed. To further evaluate the ability of iCLIP to preserve high-resolution negative spaces, we designed and printed a bifurcating microfluidic network with a 30° pitch, varying the channel diameter from 50 µm to 200 µm. Optical micrograph images of the cross-sectional profiles of the printed microchannels confirm accurate microchannel resolution achieved by iCLIP (Fig. 3 *C* and *D*). Furthermore, each channel diameter print was bisected at the channel apex and imaged using scanning electron microscopy (SEM) (*SI Appendix*, Fig. S5).

### iCLIP Process Characterization.

To further evaluate the iCLIP process, we investigated the impact that the injection rate of fresh polymerizable resin has on channel resolution. We introduce a dimensionless turnover number (Tu) representing the ratio of injection rate to fabrication rate of negative space (the average rate of microchannel volume being printed). For a given set of print parameters, Tu quantifies the number of print layers cleared by fresh resin before subsequent UV light exposure. For instance, when the injection rate is zero, simulating a traditional CLIP print, the Tu = 0. When the injection rate matches the fabrication rate, the Tu = 1, and when the injection rate exceeds the fabrication rate, the Tu > 1. We further define the dimensionless channel diameter as d/D, where d is the resulting channel diameter measured by optical microscopy after printing and D is the designed channel diameter.

**Fig. 4.**
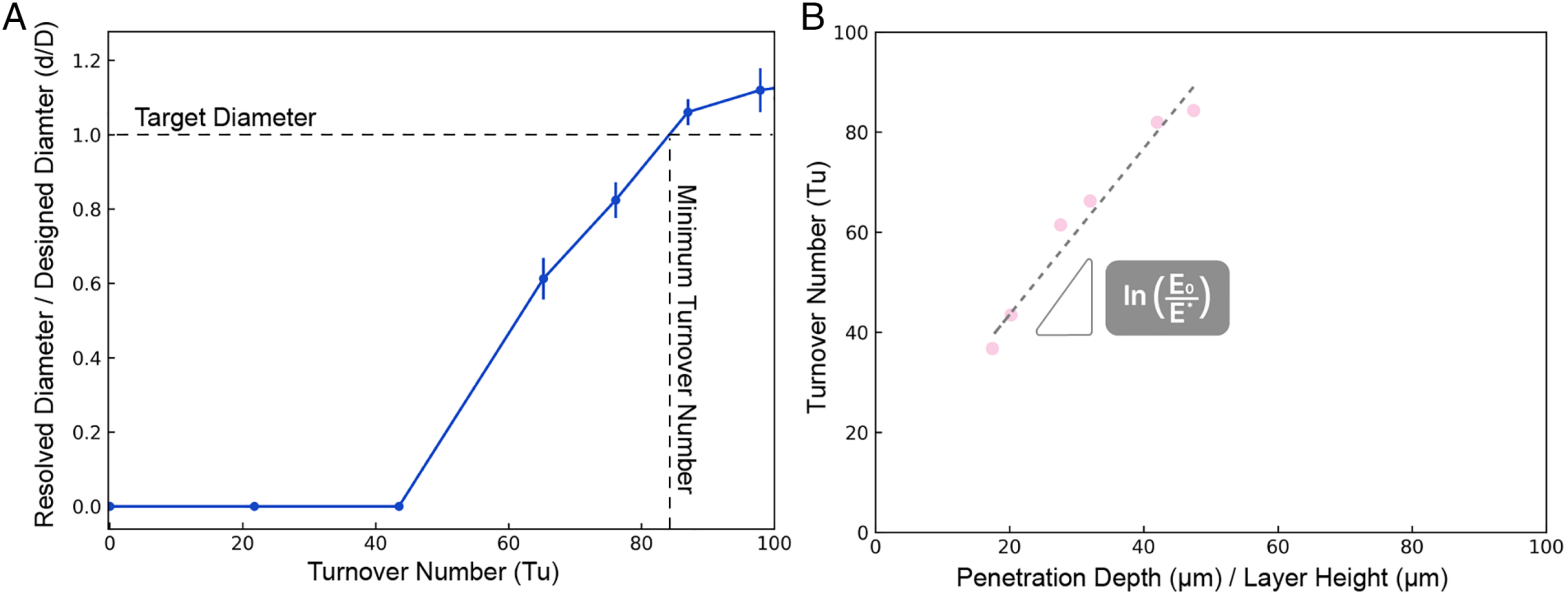
Exploring microchannel resolution in relation to fresh resin turnover. (*A*) Resolution of a resin with penetration depth of 237 μm as a function of turnover number. (*B*) Relationship between a resin’s penetration depth and the minimum required turnover rate.

Fig. 4 assesses microchannel resolution as Tu is manipulated during the printing of microfluidic structures. Traditional CLIP conditions (Tu = 0) led to unresolved channels due to overcuring. As Tu increased, d/D approached 1, indicating the minimum Tu required to resolve a given microfluidic structure. Notably, in this case, for a resin with a D_p_ of 237 µm, achieving a Tu greater than approximately 84 proved essential for precise microchannel resolution (Fig. 4*A*). Beyond the minimum Tu at exceedingly higher flow rates, channel widening beyond the designed channel diameter and, ultimately, channel cracking at extreme flow rates were observed. To further explore the impact of Tu, we investigate the dependance of D_p_ on the minimum Tu. Fig. 4*B* illustrates how the D_p_ of various resins affects the required Tu to achieve accurate negative features. To conduct this study, we examined how varying Tu influenced the ability to resolve a 100-µm bifurcating microchannel at a 30° pitch. Our observations indicated that for different resins with varying D_p_ values ranging from 65 µm to 237 µm, the corresponding minimum Tu for accurate resolution increased with higher D_p_ values. This outcome aligns with expectations because an increase in the D_p_ allows UV light to penetrate deeper into the printed part. When D_p_ is larger, UV light reaches further into the resin, exposing a larger number of previously fabricated print layers to a higher dose of UV light. Consequently, a greater penetration depth necessitates more resin displacement with each fabricated layer to prevent overcuring and ensure proper layer formation in the microfluidic structures created earlier in the print. To avoid the accumulation of excess UV energy, the iCLIP system must flow higher volumes of resin through the microfluidic structure to maintain the desired resolution. We formulated a hypothesis that precise channel resolution requires the replacement of resin within a channel before printing each subsequent layer if the original resin had accumulated a dose exceeding a critical threshold, which we denote as E*. By following a derivation process similar to the Jacobs working curve, we expressed the relationship between Tu and threshold dose as outlined in Eq. **2** (27):

[2] $$\text{Turnover }\text{Number }\left(\text{Tu}\right)=\frac{D_{p}}{s}\text{ln}\left(\frac{E_{0}}{E^{*}}\right),$$

where E_0_ is the layer exposure dose and E* is the critical threshold dose. Fig. 4*D* shows a plot of the turnover number as a function of resin penetration depth. We observe, in accordance with Eq. **2**, the minimum turnover number required to accurately resolve negative features scales linearly with penetration depth; the slope of this line is $\frac{1}{s}\text{ln}\left(\frac{E_{0}}{E^{*}}\right)$. This analysis shows that for a given penetration depth and exposure dose during part fabrication, we can determine the minimum required resin turnover number to achieve high-resolution negative features.

## Discussion

### High-Resolution iCLIP Future Applications.

**Fig. 5.**
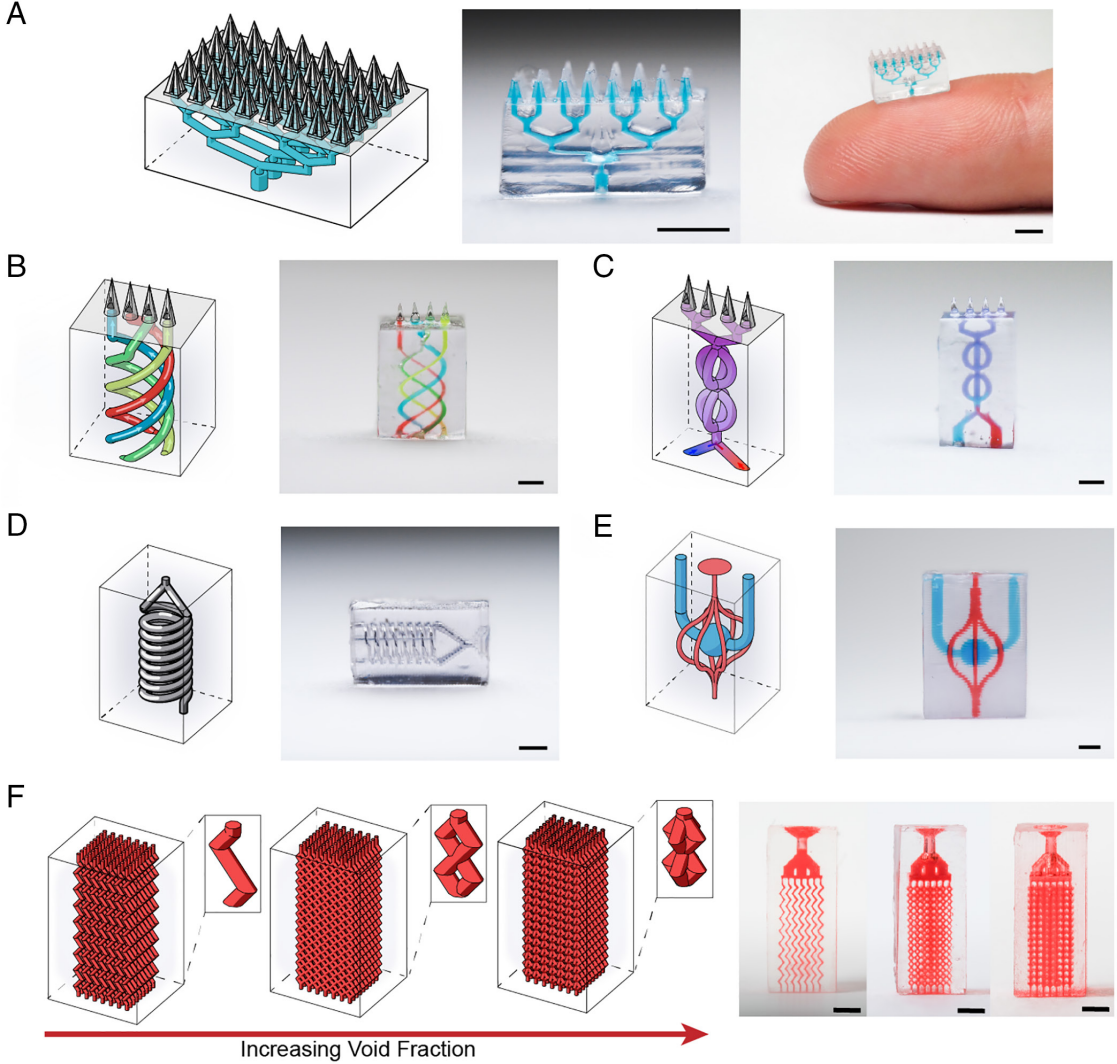
Diverse microsystem fabrication capabilities enabled by iCLIP. (*A*) Microfluidic enabled microneedle patch. (*B*) Microneedle patch with interconnected microfluidic channels. (*C*) Microfluidic inductor back-filled with conductive gallium. (*D*) Microfluidic microneedle patch with 3D micromixer. (*E*) Vascular perfusion chamber. (*F*) Porous media separation columns with varying void fraction unit cells. All scale bars 1 mm.

iCLIP can be utilized to fabricate free-form structures having micrometer scale feature resolution in XY and Z coordinates. This process control framework was used to construct various microsystems ranging from personalized medical technologies to microelectromechanical systems (Fig. 5).

Advances in bioengineering and material science have led to the development of personalized medical technologies that enable disease diagnostics and therapeutic delivery at the “point of person” (28, 29). Microneedles are a promising solution for transdermal drug delivery because they are minimally invasive (30). Here, we leverage the iCLIP platform 3D-print microfluidic elements with microneedle technology in a biologically compatible resin to provide new fluid management capabilities for transdermal drug delivery and unique fill-finish opportunities of such devices (Fig. 5 *A*–*C*) (31). Also, 3D printing technologies are becoming increasingly attractive in the manufacture of microelectromechanical systems because of their ability to fabricate free-form design geometries. We also leveraged the iCLIP process to construct 3D microstructures with embedded gallium metal conductive elements such as a 3D-printed inductor (Fig. 5*D*). Furthermore, iCLIP can fabricate interlocking vascular perfusion networks for molecular blood transport systems as shown in Fig. 5*E*. Finally, beyond microchannels and voids, the iCLIP system has shown the ability to print porous perfusion networks to perform enhanced separations (Fig. 5*F*).

We introduce iCLIP as a 3D printing method that enables the free-form fabrication of microsystems that uses a fluid control methodology rather than use of optical dyes. This approach can resolve microscale negative spaces breaking the historical relationship between resin penetration depth and negative feature resolution. This process breaks traditional resolution capabilities opening the ability to print high-resolution microsystems in materials and designs not before possible. Furthermore, the iCLIP process could be used to inject many different classes of displacing agents including nonpolymerizable fluids such as water and air (which need to be delicately controlled to avoid bleed through into the active polymerization region of the dead zone) to allow for even higher-resolution negative spaces.

## Materials and Methods

### Materials.

The resins used in the experiments are commercially available resins including Autodesk Standard Clear Prototyping Resin (PR-48; Autodesk, CA), KeySplint Hard Resin (Keystone Industries, NJ), and Whip Mix Surgical Guide Resin (Whip Mix, KY). These experimental resins were chosen because they are colorless and have penetration depths ranging from 65 μm to 237 μm.

### Resin Formulations.

D_p_—237 μm—100 wt.% Whip Mix Surgical Guide Resin.

D_p_—210 μm—95 wt.% Whip Mix Surgical Guide Resin and 5 wt.% Autodesk Standard Clear Prototyping Resin (PR-48).

D_p_—160 μm—85 wt.% Whip Mix Surgical Guide Resin and 15 wt.% PR-48.

D_p_—138 μm—75 wt.% Whip Mix Surgical Guide Resin and 25 wt.% PR-48.

D_p_—101 μm—100 wt.% KeySplint Hard Resin.

D_p_—87 μm—45 wt.% Whip Mix Surgical Guide Resin and 55 wt.% PR-48.

D_p_—65 μm—100 wt.% PR-48.

To visualize the channel geometry, negative spaces were filled with UMA 90 White Resin (Carbon3D, CA). This resin was further colored with the addition of Alumilite Flo Red, Alumilite Flo Blue, Alumilite Flo Green, and/or Alumilite Flo Yellow resin dye (Alumilite, MI). Gallium metal was used to fill microstructures with a conductive element (Amazon, WA). Isopropyl alcohol (IPA, 99%), sourced from Fisher Scientific (MA), served as the rinsing solvent for cleaning each printed sample.

### Equipment.

All experiments were performed on the high-resolution injection CLIP printer. The high-resolution injection CLIP 3D printer’s hardware can be segmented into four main components (*SI Appendix*, Fig. S1):

(i) Optical projection components. A light engine operating at wavelength 385 nm (3DLP9000, Digital Light Innovations, TX), along with a Collimation Adapter (SM2F, Thor Labs, NJ), Tube Lens (TTL200, Thor Labs, NJ), Cage Cube (CM1-DCH, Thor Labs, NJ), 4.8-μm Pixel Objective (TL2X-SAP, Thor Labs, NJ) were used in this set up. The pixel resolution of this printer was 4.8 μm.
(ii) Oxygen-permeable resin vat. A custom-designed 3D-printed build window with an oxygen-permeable window was used (Random Technology, CA). Teflon AF2400 film was used as it allows UV to penetrate through for photopolymerization and allows oxygen to permeate through the build window to inhibit photopolymerization establishing the dead zone. The dead zone is a thin layer which ranges from 50-μm to 100-μm thick.
(iii) The Build Platform. For precise vertical adjustments, a high-precision vertical translation stage (GTS70V, Newport, CA) is employed. Affixed to this translational stage is a tailor-made 3D-printed build platform and an SEM mount (Ted Pella Inc., Redding, CA) which was used as a build platform onto which 3D-printed parts were printed. The SEM mount was further machined to include a 1-mm injection port centered on the build area.
(iv) Syringe pump. A high-precision syringe pump (PHD ULTRA 4400, Harvard Apparatus, MA) was used to inject a controlled amount of fresh resin through the build platform to eliminate print-through in negative spaces.

The high-resolution iCLIP 3D printer is operated using a custom software application called CLIP3DGUI. This application is developed in Qt Creator (Qt Creator, Finland) using C++. Through user inputted parameters the CLIP3DGUI manages the operation of the UV light engine and the translation stage.

### 3D Design and Printing Process.

3D CAD objects were designed using Autodesk Fusion (Autodesk, CA). The 3D CAD models were then transferred to Autodesk Netfabb and each CAD model was sliced using a 5 μm layer height to create a series of one-bit binary image slices measuring 2,560 × 1,600 pixels.

These images were then processed using the CLIP3DGUI. This program encodes the one-bit binary images into 24-bit RGB images, resulting in 24 one-bit images being consolidated into a single frame. The encoded images were then streamed to the light engine via an HDMI cable.

The print process was controlled as follows. To ensure bonding between the build platform and the 3D part, the initial exposure dose was set to 10 times the minimum required exposure dose to polymerize the first slice layer. After the initial exposure layer, the injection of fresh resin through the build platform and into the negative space being fabricated was started at a set flow rate. The remainder of the print layers were exposed to 1.25 times the minimum required exposure dose to polymerize each 5-μm layer. Upon print completion, the injection of fresh resin is stopped, and the 3D part is cleaned. To clean the final part, isopropyl alcohol was used as the rinsing solvent to clean each printed sample. Specifically, each feature was flushed with isopropyl alcohol until no uncured resin remained in the print. The objects were then placed in an APM LED UV Cube for 10 min (APM Technica, Switzerland).

Finally, each part was processed and imaged. To improve the surface finish, a thin surface coat of OPI nature strong, high-shine, nail lacquer was applied to each print surface (OPI, CA). To evaluate the channel resolution in Main Text Fig. 4 *A*–*D*, an Olympus DSX Digital Microscope (DSX10-UZH) was used with a 3× objective lens (DSX10-SXLOB) (Evident Scientific, CA). The channel diameter was measured to determine negative space resolution. To image the resultant prints in Main Text Figs. 1, 2, and 5, an Olympus OM-D Camera was used, coupled with an Olympus M.Zuiko Digital ED 60-mm 1:2.8 Macro lens (Olympus, Japan).

### Minimum Turnover Number.

The ability to resolve an accurate channel structure is dependent on the resin turnover, or turnover number (Tu) during part fabrication. Specifically, the minimum Tu was determined experimentally by increasing the Tu until the printed microfluidic structure matched the designed structure. Using Main Text Fig. 4 and *SI Appendix*, Fig. S5, we identified where the line graph intersects or crosses the normalized diameter value of 1, indicating the minimum Tu. This specific value was chosen because it signifies at this turnover number the channel has been entirely resolved, aligning with the intended design specification. By selecting the minimum turnover number at this juncture, the design specification is effectively met, and any further optimization might not be necessary since the channel has already achieved its desired outcome.

## Supplementary Material

Appendix 01 (PDF)

## Data Availability

All study data are included in the article and/or *SI Appendix*.
