# Supplementary material for: High-resolution stereolithography: Negative spaces enabled by control of fluid mechanics
Source: Proc Natl Acad Sci U S A. 2024 Sep 4;121(37):e2405382121. doi: 10.1073/pnas.2405382121 (PMC11406279; doi:10.1073/pnas.2405382121)
Supplement: Supplementary file 1 — Appendix 01 (PDF) [file pnas.2405382121.sapp.pdf]

PNAS

Supporting Information for
High-Resolution Stereolithography: Negative Spaces Enabled by Control of Fluid Mechanics

Ian A. Coates[a], William Pan[b], Max A. Saccone[a,c], Gabriel Lipkowitz[b], Dan Ilyin[b], Madison M. Driskill[a], Maria T. Dulay[c], Curtis W. Frank[a], Eric S. G. Shaqfeh[a,b], Joseph M. DeSimone[a,c,1]

[a] Department of Chemical Engineering, Stanford University, Stanford, CA 94305
[b] Department of Mechanical Engineering, Stanford University, Stanford, CA 94305
[c] Department of Radiology, Stanford University, Stanford, CA 94305
[1] To whom correspondence may be addressed. Email: jmdesimone@stanford.edu

This PDF file includes:

Supporting text
Figures S1 to S6
SI References

Supporting Information Text

Measuring UV Penetration Depth of Resins

To ascertain the depth to which UV light penetrates a series of resins, a working curve calibration was performed. To identify the penetration depth and critical exposure energy the Beer-Lambert relationship, referenced in Main Text Equation 1, is used:

$$\text{Equation S1: } E_n = t\, I_0\, e^{-sz/D_p}$$

Where n is the number of layers from the dead zone, I_0 is the intensity of the UV irradiation at the dead zone, t is the UV exposure time, s is the layer slice thickness of each exposure, and D_p is the penetration depth (1–4).

The Beer-Lambert relationship can be rearranged to establish the Jacobs' working curve equation (5):

$$\text{Equation S2: } C_d = D_p \ln\left(\frac{E_0}{E_c}\right)$$

Where Cd is the cure depth, E_0 is the UV dose energy delivered, and E_c is the critical UV dose energy to cause polymerization at a corresponding cure depth, C_d.

Using Jacob's working curve equation, a set of resin dots was subjected to different levels of UV dose energy. Specifically, a thin glass slide was placed on the printer build window. The resin of interest was then placed on the glass slide. After which each resin dot was exposed to a varying UV dose energy. The cured thickness of each resin dot was measured using a Mitutoyo 547-500S Height Gauge (Mitutoyo, IL) (Fig. S2). To determine the corresponding UV penetration depth (D_p) and critical dose energy (E_c), the cured thickness of each resin dot and the respective logarithm of UV dose were plotted and fitted using a linear least-squares solver. From the fit parameters the D_p and E_c of each tested resin were determined (Fig. S2).

UV Light Accumulation Model

The accumulated dosage in an arbitrary part geometry was determined by calculating the total UV dose each individual voxel receives after each exposure and all subsequent exposures. Given any geometry designed in a computer-aided design tool, we sliced the part into png files along the Z-axis using Autodesk Netfabb 2023 with a layer height of 5 µm and an XY resolution of 4.8 µm pixels. Each slice was represented with black and white pixels to represent exposed and unexposed voxels, respectively. The slices were then sequentially imported into a Python script which calculated the accumulated dosage at each voxel based on the Beer-Lambert relation as referenced in Main Text Equation 1 (4, 6, 7):

$$\text{Equation S3: } E_n = \sum_{z=0}^{n} I_o t\, e^{-sz/D_p}$$

where n is the number of layers from the dead zone, I_0 is the intensity of the UV irradiation at the dead zone, t is the UV exposure time, s is the layer slice thickness of each exposure, and D_p is the penetration depth determined by the resin's material properties at the UV wavelength 385 nm.

By using open-source libraries such as OpenCV 2 and NumPy, we created a binary 3D array, A, with a shape of (X, Y, Z) to represent each voxel where the integer 1 represented an exposed voxel, and 0 represented an unexposed voxel. To simplify the calculation without having to iterate over every layer with every subsequent layer, we can create a lower left triangular matrix XD with a shape of (Z, Z):

$$\chi_D = \begin{bmatrix} d_1 & 0 & 0 & \cdots & 0 & 0 \\ d_2 & d_1 & 0 & \cdots & 0 & 0 \\ d_3 & d_2 & d_1 & \cdots & 0 & 0 \\ \vdots & \vdots & \vdots & \ddots & \vdots & \vdots \\ d_{n-1} & d_{n-2} & d_{n-3} & \cdots & d_1 & 0 \\ d_n & d_{n-1} & d_{n-2} & \cdots & d_2 & d_1 \end{bmatrix}$$

where E_n is the dose energy n layers away from the dead zone:

Equation S5: $E_n = I_o t e^{-sn/D_p}$

We then further indexed 2D arrays A_{xz} or A_{yz} with a shape of (X, Z) and (Y, Z), respectively, to get the accumulated dosage matrix Δn of every voxel in the XZ or YZ planes at a specific build layer n:

Equation S6: $\Delta_{\mathrm{n,xz}} = \mathrm{A_{xz}} \times \chi_{\mathrm{D}}$

Equation S7: $\Delta_{n,\mathrm{yz}} = A_{yz} \times \chi_{\mathrm{D}}$

After calculation of the final dose at each pixel in the X, Y, and Z directions all voxels in designed negative space which exceeded the critical polymerization energy, $E_n > E_c$ for a specified layer height, were colored blue to represent overcuring and voxels which remained uncured, or a voxel which $E_n < E_c$ for a specified layer height, remained white.

3D Prints

Main Text Figure 1: The prints identified in Figure 1C were printed using PR-48 resin, KeySplint Hard resin, and Whip Mix Surgical Guide resin. The evaluation of these channels can be found in Figure S4A and S4C, Figure 3B and 3D, and Figure S4B and S4D, respectively. The prints in Figure 1D were printed utilizing KeySplint Hard resin.
Main Text Figure 2: The sinuous microstructure was printed using KeySplint Hard resin. Under CLIP conditions (Figure 2A), the injection rate was set to 0 µL/min. In contrast, under iCLIP conditions (Figure 2B), the injection rate of KeySplint Hard resin was set to 5 µL/min.
Main Text Figure 3: All microfluidic channels were printed using KeySplint Hard. For CLIP conditions, the injection rate remained at 0 µL/min. Conversely, under iCLIP conditions, the injection rate was set at 1.1 times the minimum turnover number.
Main Text Figure 4: All microfluidic channels were printed using Whip Mix Surgical Guide resin.
Main Text Figure 5: Main Text Figure 5A – 5E were printed with KeySplint Hard. The injection rate of Figure 5A was set to 4 µL/min. The injection rate for Figure 5B-5E was set to 2.5 µL/min. Figure 5F was printed with PR-48 resin. The injection rate was set to 2 µL/min.

Derivation of Turnover Number

The iCLIP process requires displacing trapped resin in negative spaces with fresh resin to prevent overcuring. Here we present a derivation to relate the required resin turnover with UV penetration depth of a print resin (*5*).

In this case, the turnover number can be defined as the volume of negative space within a certain number of layers which must be displaced before a subsequent exposure can occur. Specifically, the turnover number (Tu) can be represented by the following:

Equation S8: $Tu = \frac{z}{s}$

where *z* is the vertical layer height required to be flushed and *s* is the print layer height. Using this relationship, we hypothesize that we need to reach a resin turnover number high enough to flush

all resin out of a negative space that has accumulated a dose, *E*, larger than the critical flush dose, E^*.

Adapting Beer-Lambert's Law, we are given the expression:

$$\text{Equation S9: } E(z = s * Tu) = E^* = E_0 e^{\frac{-s*Tu}{D_p}}$$

where E_0 is the exposure energy dose at z=0 and D_p is the UV penetration depth of a resin. Ultimately using this relationship, we define *Tu* as a function of the UV penetration depth of a resin, layer height, and exposure dose.

$$\text{Equation S10: } Tu = \frac{D_p}{s} \ln\left(\frac{E_0}{E^*}\right)$$

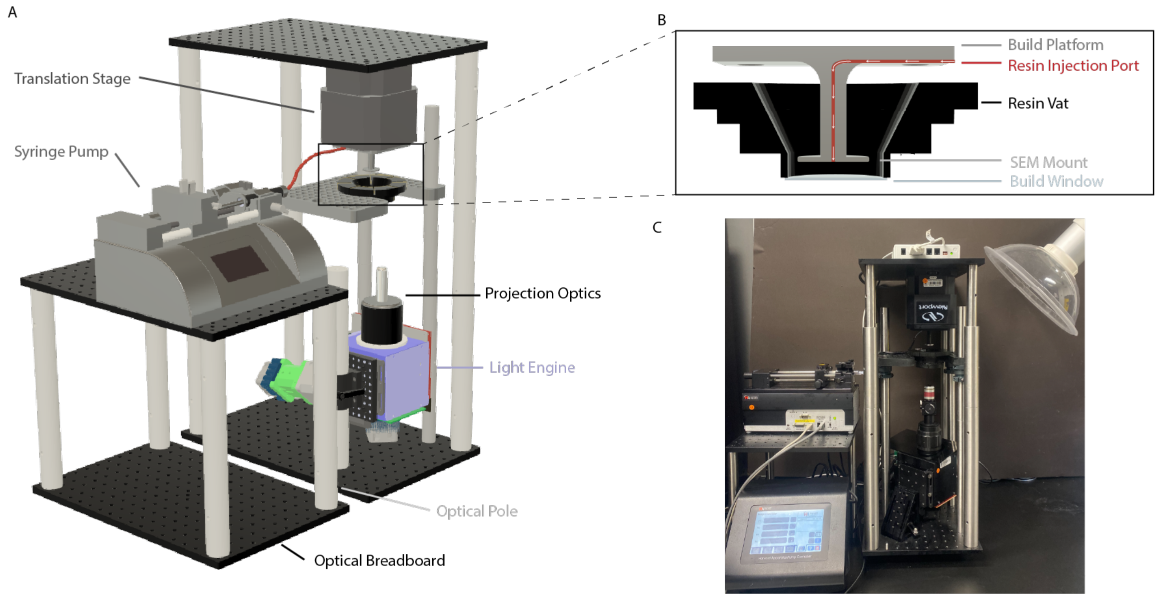

Fig. S1. High Resolution iCLIP printer. (A) iCLIP printer schematic. **(B)** iCLIP build platform schematic. **(C)** iCLIP printer.

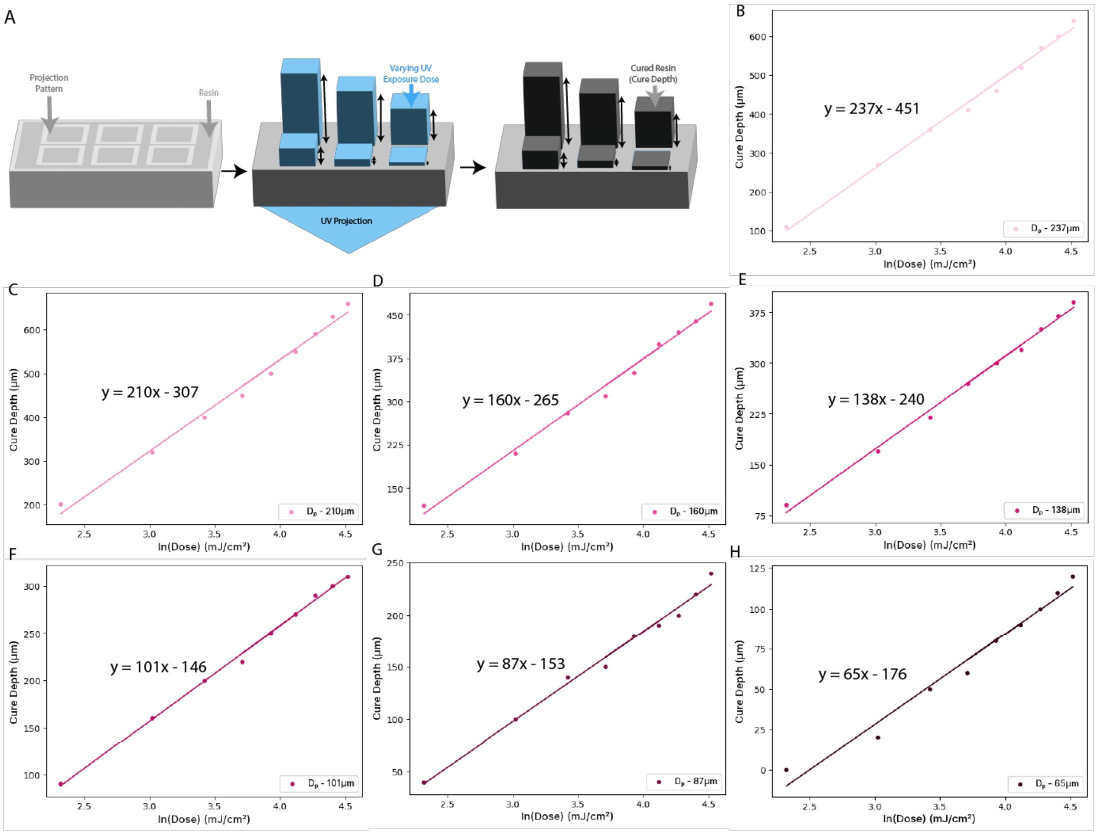

Fig. S2. Measuring penetration depth. (A) Measuring penetration depth schematic. **(B)** Working Curve Calibration of resin with D_p 237 µm. **(B)** Working Curve Calibration of resin with D_p 237µm. **(C)** Working Curve Calibration of resin with D_p 237 µm. **(D)** Working Curve Calibration of resin with D_p 210 µm. **(E)** Working Curve Calibration of resin with D_p 160 µm. **(F)** Working Curve Calibration of resin with D_p 138 µm.**(G)** Working Curve Calibration of resin with D_p 101 µm. **(H)** Working Curve Calibration of resin with D_p 87 µm. **(I)** Working Curve Calibration of resin with D_p 65 µm.

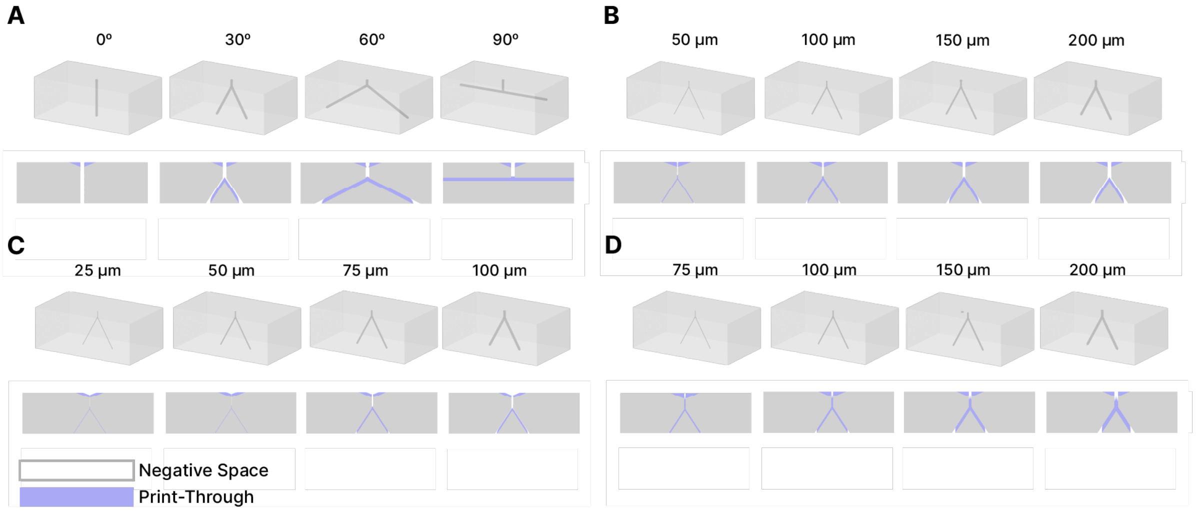

Fig. S3. UV accumulation energy in microchannel designs. (A) UV accumulation energy model in resin with D_p 101 μm in varying channel geometries. **(B)** UV accumulation energy model in resin with D_p 101 μm in varying channel diameters. **(C)** UV accumulation energy model in resin with D_p 65 μm in varying channel diameters. **(D)** UV accumulation energy model in resin with D_p 237 μm in varying channel diameters.

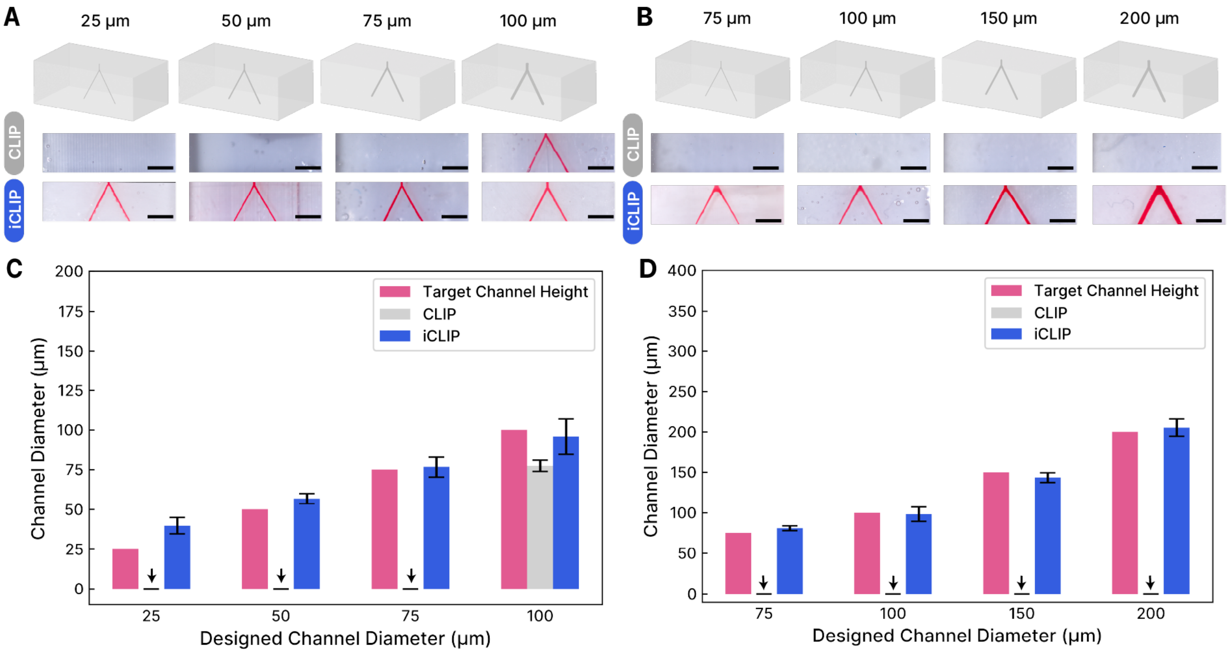

Fig. S4. Mitigating overcuring in varying microfluidic channel sizes with varying resins. (A) Resulting CLIP and iCLIP prints of varying channel diameters in resin with D_p 65 µm. **(B)** Resulting CLIP and iCLIP prints of varying channel diameters in resin with D_p 237 µm. **(C)** Evaluating resolution of varying channel diameters printed with resin D_p 65 µm using the CLIP and iCLIP systems. **(D)** Evaluating resolution of varying channel diameter printed with resin D_p 237 µm using the CLIP and iCLIP systems.

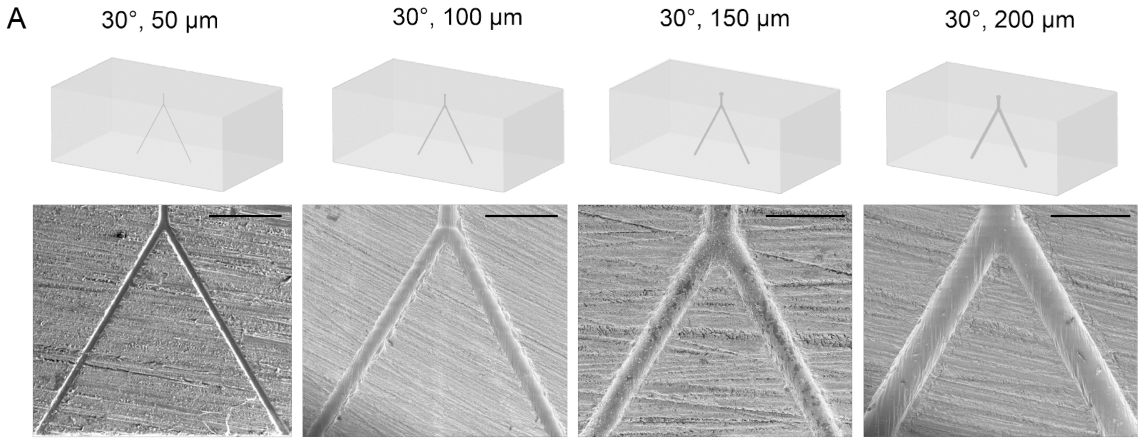

Figure S5. Mitigating overcuring in varying microfluidic channel geometries and sizes. (A) Resulting CLIP and iCLIP prints of varying channel diameters from 50 μm to 200 μm. All scale bars 500 μm.

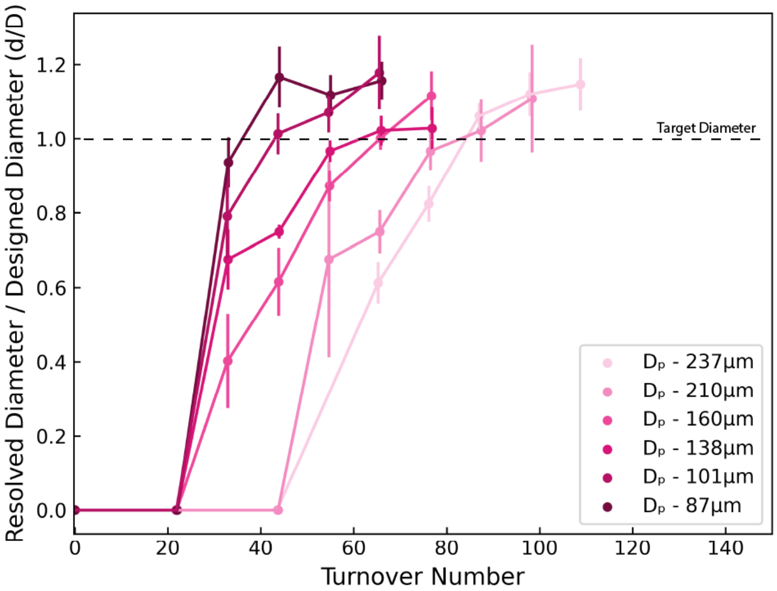

Fig. S6. Resolution of microfluidic channels printing with resins of varying penetration depths as a function of turnover number.

SI References

1. A. Champion, B. Metral, A. Schuller, C. Croutxé-Barghorn, C. Ley, L. Halbardier, X. Allonas, A Simple and Efficient Model to Determine the Photonic Parameters of a Photopolymerizable Resin Usable in 3D Printing. *ChemPhotoChem*. **5**, 839–846 (2021).
2. Y. Wang, Y. Wang, C. Mao, D. Mei, Printing depth modeling, printing process quantification and quick-decision of printing parameters in micro-vat polymerization. *Materials & Design*. **227**, 111698 (2023).
3. H. Gong, M. Beauchamp, S. Perry, A. T. Woolley, G. P. Nordin, Optical approach to resin formulation for 3D printed microfluidics. *RSC Adv.* **5**, 106621–106632 (2015).
4. Y. Xu, F. Qi, H. Mao, S. Li, Y. Zhu, J. Gong, L. Wang, N. Malmstadt, Y. Chen, In-situ transfer vat photopolymerization for transparent microfluidic device fabrication. *Nat Commun*. **13**, 918 (2022).
5. P. F. Jacobs, Fundamentals of Stereolithography. *Society of Manufacturing Engineers*. 196-211 (1992).
6. H. Gong, B. P. Bickham, A. T. Woolley, G. P. Nordin, Custom 3D printer and resin for 18 μm × 20 μm microfluidic flow channels. *Lab Chip*. **17**, 2899–2909 (2017).
7. Z. D. Pritchard, M. P. Beer, R. J. Whelan, T. F. Scott, M. A. Burns, Modeling and Correcting Cure-Through in Continuous Stereolithographic 3D Printing. *Adv. Mater. Technol.* **4**, 1900700 (2019).
